# Healing pattern classification for thoracolumbar burst fractures after posterior short-segment fixation

**DOI:** 10.1186/s12891-020-03386-z

**Published:** 2020-06-12

**Authors:** Changxiang Liang, Guihua Liu, Guoyan Liang, Xiaoqing Zheng, Dong Yin, Dan Xiao, Shixing Zeng, Honghua Cai, Yunbing Chang

**Affiliations:** 1 Spine departement, Orthopedic center, Guangdong Provincial People’s Hospital (Guangdong Academy of Medical Sciences), 510080, No.106, Zhongshan 2nd Road, Guangzhou, Guangdong Province China; 2 Orthopedic department, Huizhou Municipal Central Hospital, Huizhou City, China

**Keywords:** Healing pattern classification, Vertebral cavity, Thoracolumbar fracture, Burst fracture, Stability

## Abstract

**Background:**

Thoracolumbar burst fractures can be treated with posterior short-segment fixation. However, no classification can help to estimate whether the healed vertebral body will have sufficient stability after implant removal. We aimed to develop a Healing Pattern Classification (HPC) to evaluate the stability of the healed vertebra based on cavity size and location.

**Methods:**

Fifty-two thoracolumbar burst fracture patients treated with posterior short-segmental fixation without fusion and followed up for an average of 3.2 years were retrospectively studied. The HPC was divided into 4 types: type I - no cavity; type II - a small cavity with or without the violation of one endplate; type III - a large cavity with or without the violation of one endplate; and type IV - a burst cavity with the violation of both endplates or the lateral cortical shell. The intraobserver and interobserver intraclass correlation coefficients (ICCs) of the HPC were assessed. The demographic characteristics and clinical outcomes of the cohort were compared between the stable group (types I and II) and the unstable group (types III and IV). Logistic regression was conducted to evaluate risk factors for unstable healing.

**Results:**

The intraobserver and interobserver ICCs of the HPC were 0.86 (95% CI = 0.74–0.90) and 0.77 (95% CI = 0.59–0.86), respectively. While the unstable healing group (types III and IV) accounted for 59.6% of the patients, most of these patients were asymptomatic. The preoperative Load Sharing Classification (LSC) comminution score may predict the occurrence of unstable healing (OR = 8.4, 95% CI = 2.4–29.7).

**Conclusions:**

A reliable classification for assessing the stability of a healed vertebra was developed. With type I and II healing, the vertebra is considered stable, and the implant can be removed. With type III healing, the vertebra may have healing potential, but the implant should not be removed unless type II healing is achieved. With type IV healing, the vertebra is considered extremely unstable, and instrumentation should be maintained. Assessing the LSC comminution score preoperatively may help to predict unstable healing after surgery.

## Background

Burst fractures are characterized as failure under compression of both the anterior and middle columns [1, 2]. Thoracolumbar burst fractures at either or both endplates with the integrated posterior ligamentous complex, which are morphologically classified as type A3 or A4 by the AOSpine Classification, can be treated with posterior short-segment fixation without fusion [3–5]. Although this approach is widely accepted with satisfying outcomes, several studies found the vertebral body to recollapse and kyphosis to recur after surgery, especially after implant removal [6, 7]. Therefore, whether the implant should be removed after vertebral healing is controversial in the context of this nonfusion surgery [8–10]. The decision for implant removal is not easy to make because there are no classification systems or criteria that can help to estimate whether the healed vertebral body will have sufficient strength after implant removal.

In our experience, although most of the patients experienced fracture union after surgery, not all the fractured vertebrae healed perfectly. Cavities or lesions can remain in the healed vertebral bodies, as observed in figures from previously reported studies in the literature [9, 11–13]. Given that focal regions of bone loss have been proven to reduce the structural competence of vertebrae, these cavity lesions may be significantly related to the recurrence of vertebral collapse after implant removal [14–16]. Furthermore, the impact of a lesion on the structural properties of the vertebral body is related to its size and location [16]. Accordingly, assessing the size and location of cavities in vertebrae may help to predict vertebral stability after implant removal.

To the best of our knowledge, there are no fracture healing classification systems regarding the vertebral cavities being reported. Here, we developed a new classification, the Healing Pattern Classification (HPC), to estimate the stability of the healed vertebra based on cavity size and location. We further explored the risk factors related to unstable healing.

## Methods

### Patients

We retrospectively analyzed the prospectively collected data of consecutive patients with thoracolumbar burst fractures from 2014 to 2018 at a single hospital. The inclusion criteria were as follows: age between 18 and 60; diagnosis of acute traumatic thoracolumbar single-segmental burst fracture; AOSpine Classification type A3 or A4 [3]; surgical management with posterior short-segment fixation without fusion; more than 1 year of follow-up; and no implant removal until the last follow-up. Patients with fracture-dislocation, multiple life-threatening injuries, previous neurological diseases (stroke, etc.) that may affect the evaluation of the neurological outcome, severe osteoporosis (T-score by bone mineral densitometry of < − 3.0) [17, 18], and missing radiological data, were excluded. The Institutional Ethics Committee of the Guangdong Provincial People’s Hospital approved this study. The design and reporting of this study followed the Strengthening the Reporting of Observational Studies in Epidemiology (STROBE) statement [19].

### Surgical technique

All patients were surgically treated within 1 week after injury. The surgery was conducted with the standard technique, as previously described [4]. Briefly, patients were positioned with hyperextension of the thoracolumbar junction. Four pedicle screws were inserted into the vertebrae cephalad and caudal to the fractured vertebra. Two polyaxial screws were inserted into both pedicles of the fractured vertebra as intermediate screws [20, 21]. The intermediate screw heads were left slightly protruding to act as a push point and to achieve the reduction of kyphosis. Kyphosis was corrected through postural reduction and rod over-contouring. The fractured height of the vertebra was restored by segmental distraction. None of the injured vertebrae underwent grafting in this study. In cases of fractures with concurrent neurological deficits or spinal canal compromise greater than 50%, laminectomy was performed. Patients were allowed to walk with bracing on the day after surgery. Vigorous work and activity were restricted up to 12 weeks postoperatively.

### Data collection

Preoperative data, including sex, age, AOSpine classification, fracture location, American Spinal Injury Association (ASIA) spinal cord impairment scale, Load Sharing Classification (LSC) score and LSC subscores [22], as well as whether spinal canal decompression was performed, were collected. X-ray, computed tomography (CT) or magnetic resonance (MR) scans were obtained before the surgery and at the final follow-up. Clinical outcomes were evaluated in the out-patient department using the ASIA impairment scale, the Oswestry Disability Index (ODI) [23] and the ten-point itemized visual analog scale (VAS) [24] for low back pain at the final follow-up.

### HPC

**Fig. 1:**
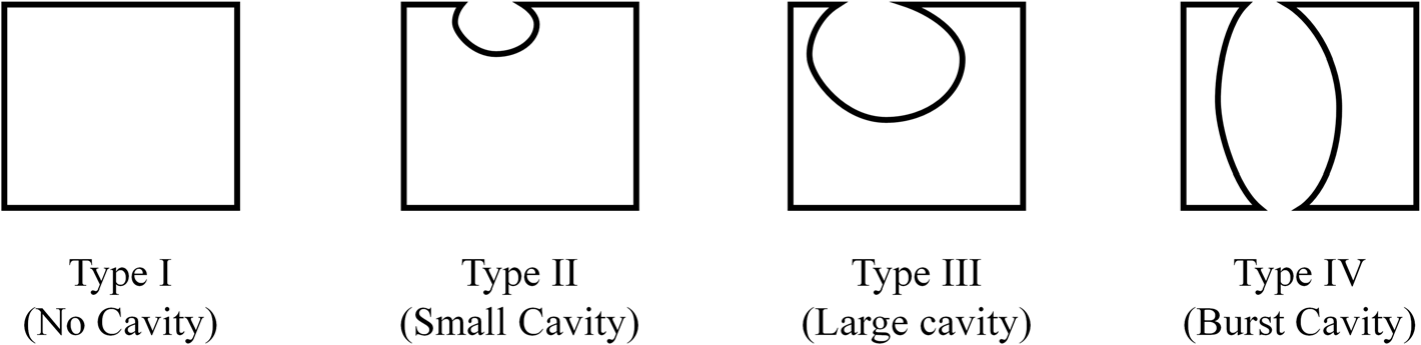
Rationale of the four healing types in the Healing Pattern Classification after posterior short-segment fixation without fusion

**Fig. 2:**
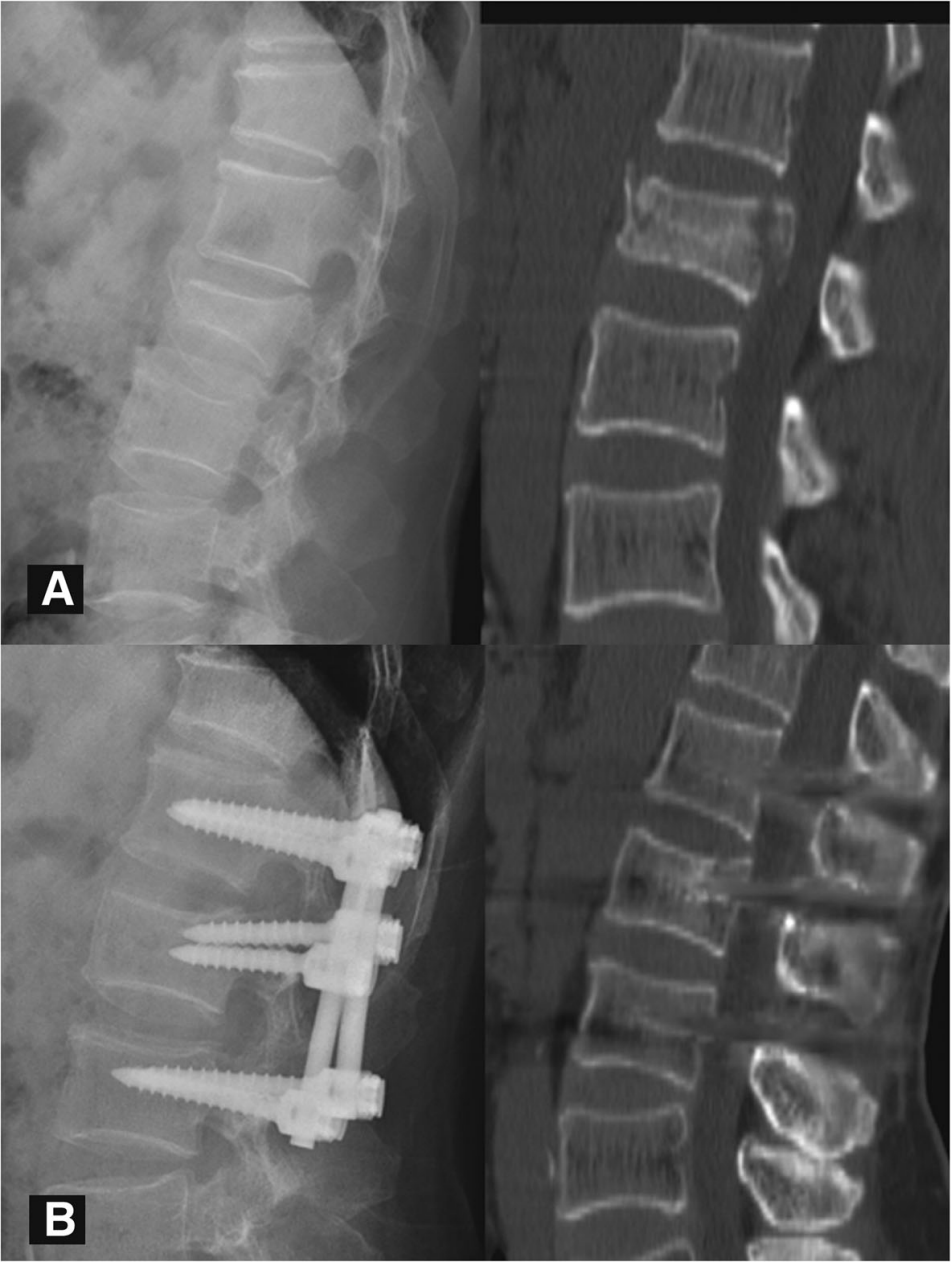
A 53-year-old male had a burst fracture at L2, with an ASIA grade for neurological function of E on admission. **a** Preoperative X-ray and CT images show an A3 burst fracture. **b** X-ray and CT images at 15 months after surgery show complete healing of the vertebral body without a cavity, which was classified as HPC type I

**Fig. 3:**
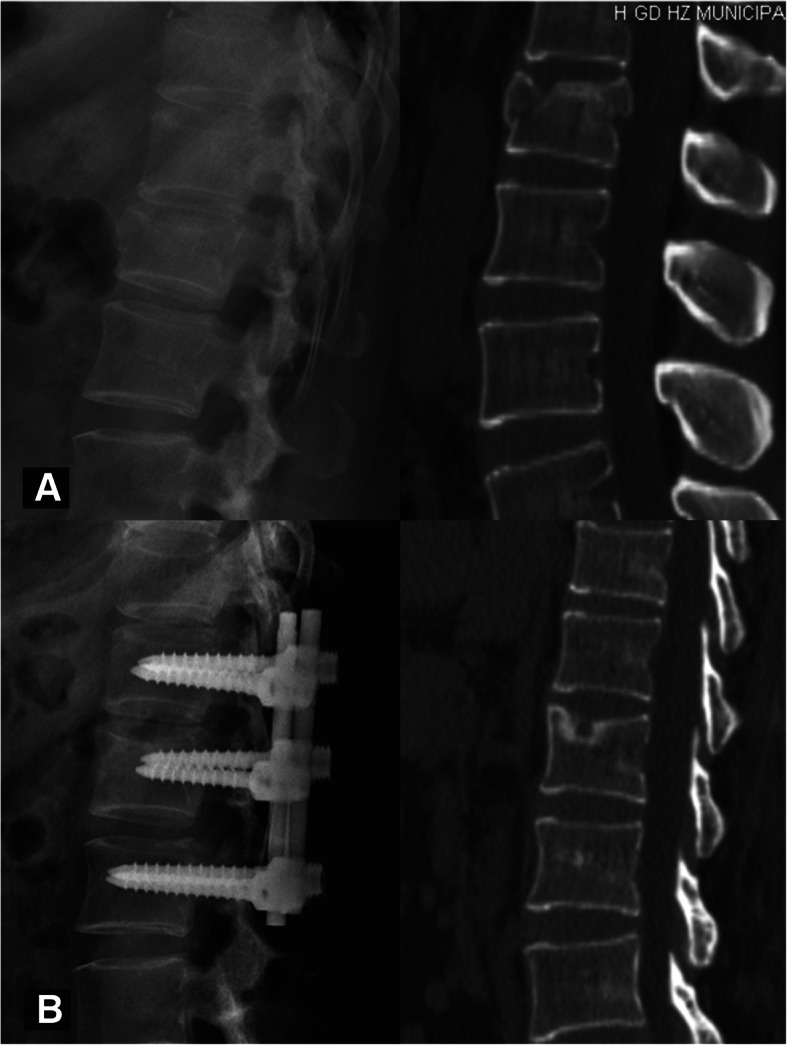
A 48-year-old female had a burst fracture at L1, with an ASIA grade for neurological function of E on admission. **a** Preoperative X-ray and CT images show an A3 burst fracture. **b** X-ray and CT images at 14 months after surgery show a defect region smaller than 1/3 of the vertebral body volume with violation of the upper endplate, which was classified as HPC type II

**Fig. 4:**
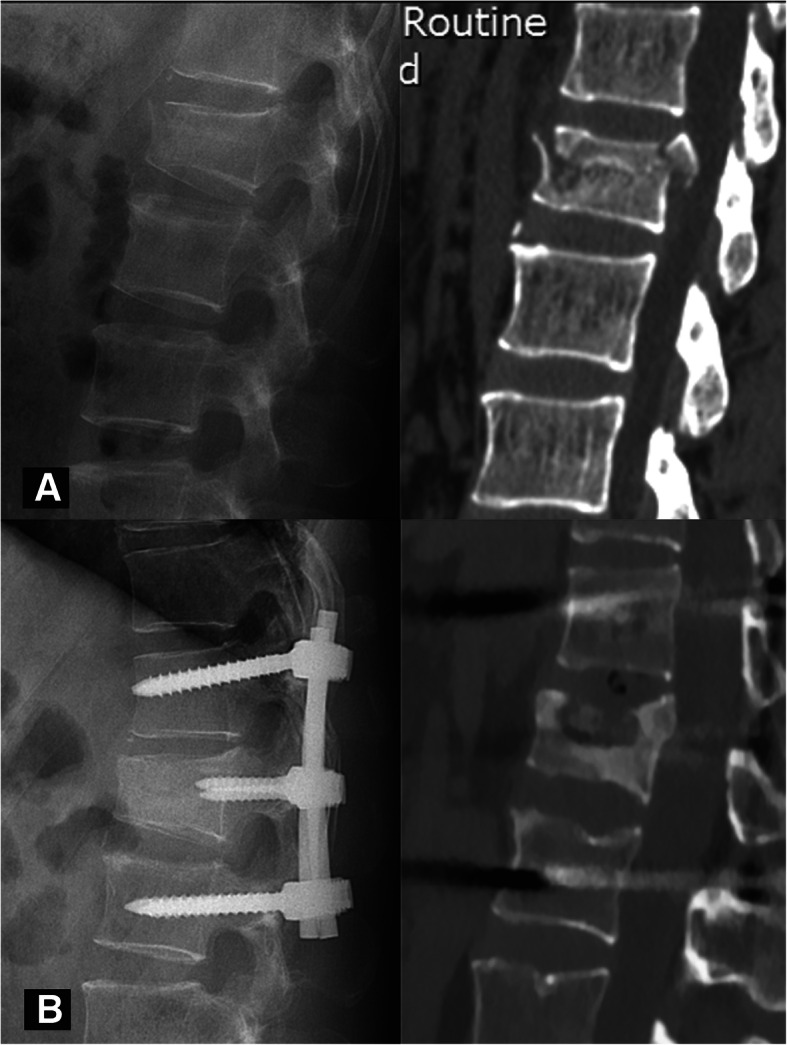
A 52-year-old male had a burst fracture at L1, with an ASIA grade for neurological function of E on admission. **a** Preoperative X-ray and CT images show an A3 burst fracture. **b** Postoperative X-ray and CT images at 18 months show a large oval cavity in the superoanterior part of the vertebral body that was larger than 1/3 of the vertebral body volume, which was classified as HPC type III

**Fig. 5:**
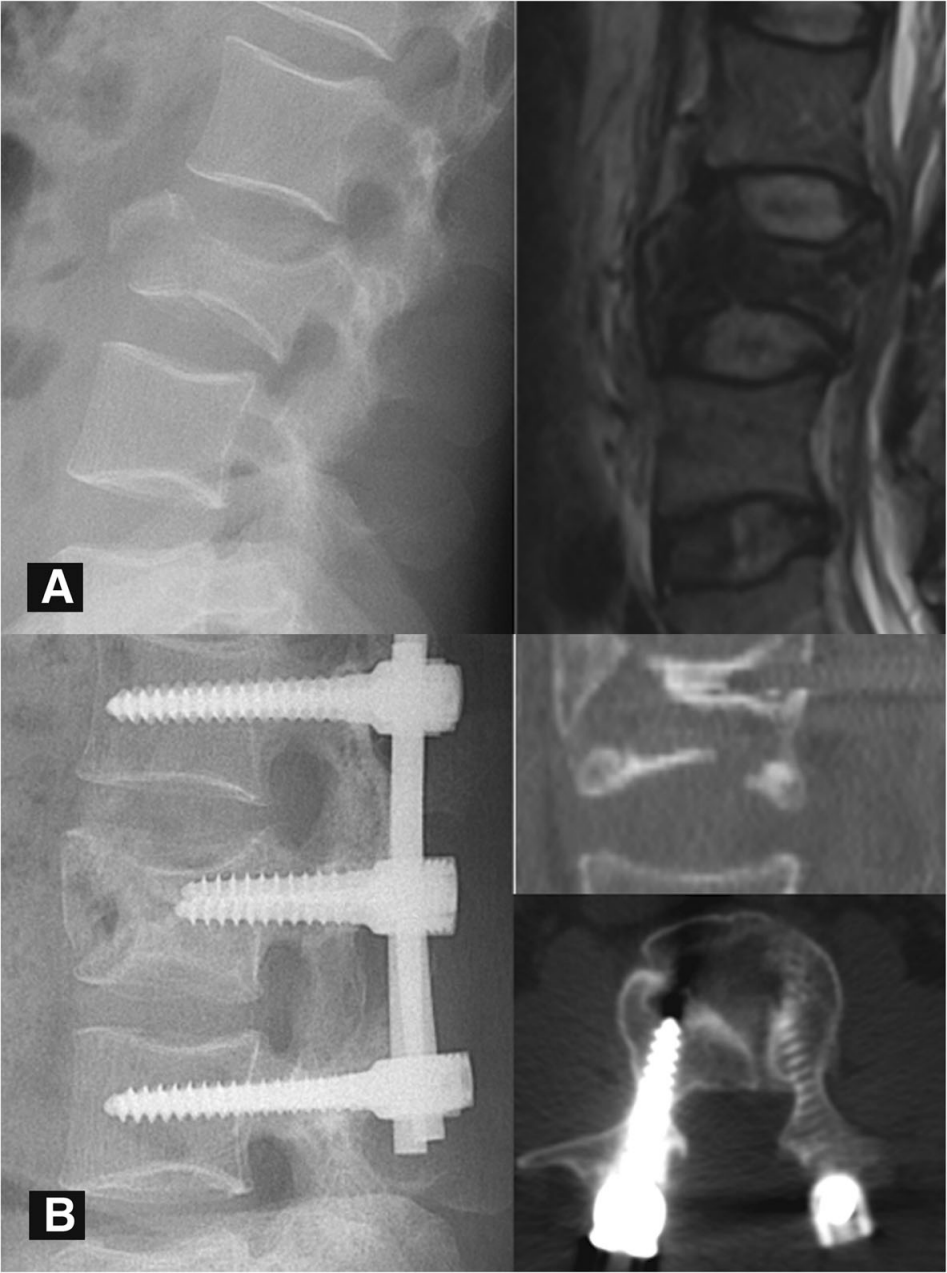
A 41-year-old male had a burst fracture at L3, with an ASIA grade for neurological function of C on admission. **a** Preoperative X-ray and MR images show an A4 burst fracture. The fracture mass protruded into the spinal canal and compressed the dura mater. **b** X-ray and CT images 18 months after surgery. The collapsed vertebra was well reduced and maintained. Several burst fragments converged into a cavity within the vertebral body, violating both endplates and the anterior vertebral shell, which was classified as HPC type IV

The rationale of the HPC is shown in Fig. 1. The healing morphology of the vertebral body was analyzed through the 3D reconstruction of CT and/or MR scans at the last follow-up. Bony union was considered a bridging of > 25% of any cross-sectional area of the fractured vertebra, despite the presence or absence of a cavity [25, 26]. A vertebra that obtained bony union was considered healed. We further defined a recognizable cavity as a hollow lesion larger than 2 mm in diameter inside the cancellous vertebral body. According to the size and location of the cavity, the healing pattern was classified into four types: type I - an intact vertebral body without any recognizable cavity (Fig. 2); type II - a small cavity (less than 1/3 of the vertebral body volume) with or without the violation of one endplate (Fig. 3); type III - a large cavity (more than 1/3 of the vertebral body volume) with or without the violation of one endplate (Fig. 4); and type IV - a burst cavity larger than 1/3 of the vertebral body volume and with the violation of both endplates or the lateral cortical shell (Fig. 5). The cut-off for a small/large cavity was set according to Costa et al.’s research, which suggests that a lytic lesion larger than approximately 1/3 of the vertebral body volume may have a significant impact on the structural properties of the vertebral body [16]. Therefore, vertebrae with HPC type I/II healing were considered stable, and those with HPC type III/IV healing were considered unstable.

### Statistical analysis

The reliability of this classification was examined by intraobserver and interobserver studies. Two residents trained in our department and two senior spine surgeons were asked to classify the type of healing outcome of the fractured vertebra. Assessments of the data were performed in random order by each observer on two separate occasions at least 5 days apart. The intraobserver and interobserver agreements of the classification were determined by the intraclass correlation coefficient (ICC), with values greater than 0.75 considered to indicate excellent agreement [27]. After reaching consistency with the HPC of each patient, we further sorted them into the stable group (types I and II) and unstable group (types III and IV). We compared the preoperative parameters and clinical outcomes of these two groups using Student’s *t*-test for continuous data and the chi-square test for categorical data to investigate the clinical manifestations of unstable healing. Spearman correlation and adjusted logistic regression were performed to assess the risk factors for unstable healing. Statistical analysis was performed with the SPSS for Windows statistical package, version 19.0 (SPSS, Chicago, Illinois). A significant difference was determined as *p* < 0.05.

## Results

### Reliability of the HPC

In total, the cases of 52 patients (36 males and 16 females) were retrieved with complete data, with a mean age of 41.5 ± 10.3 years. The median follow-up duration was 3.2 years (ranging from 12 months to 74 months). The demographic characteristics are shown in Table 1. All of the fractured vertebrae achieved bony union after surgery, and there was one case of instrumentation failure (screw breakage). Evaluations were carried out by four observers on two occasions. The intraobserver and interobserver ICCs of the HPC were 0.86 (95% CI = 0.74–0.90) and 0.77 (95% CI = 0.59–0.86), respectively. Systematic differences between observers mainly presented in cases with cavities that were approximately as large as 1/3 of the vertebral body volume; in these special cases, it may be difficult to distinguish between type II and type III. In general, these results revealed the excellent reproducibility of the HPC.

**Table 1:** Demographic Characteristics

	Stable Group			Unstable Group			***P***-value
	Type I	Type II	Total	Type III	Type IV	Total	
**Sample size**	12	9	21	19	12	31	–
**Age (mean ± SD)**	40.2 ± 11.8	47.3 ± 9.3	43.2 ± 10.9	43.7 ± 8.8	34.9 ± 9.3	40.3 ± 9.7	0.327
**Gender**							
**Male**	6	4	10	15	11	26	0.005*
**Female**	6	5	11	4	1	5	
**AOSpine Classification**							
**A3**	12	9	21	16	1	17	< 0.001*
**A4**	0	0	0	3	11	14	
**Smoking history**	2	1	3	5	5	10	0.004*
**Location of fracture**							
**T11**	0	0	0	0	1	1	0.193
**T12**	1	4	5	3	0	3	
**L1**	5	3	8	11	3	14	
**L2**	5	2	7	3	2	5	
**L3**	1	0	1	1	5	6	
**L4**	0	0	0	1	1	2	
**Load Sharing Classification score**							
**Comminution**	1.4 ± 0.7	1.7 ± 0.5	1.5 ± 0.6	2.1 ± 0.5	2.8 ± 0.5	2.3 ± 0.6	< 0.001*
**Fragmental apposition**	1.3 ± 0.7	1.2 ± 0.4	1.3 ± 0.5	1.7 ± 0.7	2.6 ± 0.7	2.1 ± 0.8	< 0.001*
**Deformity correction**	2.1 ± 0.8	2.4 ± 0.7	2.2 ± 0.7	2.5 ± 0.6	2.8 ± 0.5	2.6 ± 0.5	0.047*
**Total Score**	4.8 ± 1.7	5.2 ± 1.2	5.0 ± 1.5	6.3 ± 1.5	8.3 ± 1.1	7 ± 1.7	< 0.001*
**ASIA impairment scale**							
**B**	0	0	0	0	1	1	0.029*
**C**	0	0	0	2	2	4	
**D**	1	0	1	4	4	8	
**E**	11	9	20	13	5	18	
**Spinal canal decompression**							
**YES**	2	1	3	4	7	11	0.091
**NO**	10	8	18	15	5	20	

None of the patients had ASIA-A spinal cord impairment

*A *P*-value < 0.05 is considered statistically significant between the Stable Group and the Unstable Group

### Distribution of the four HPC types

The distribution of the four HPC types in our cohort was calculated after an agreement among all investigators was reached. The numbers and percentages of HPC types I/II/III/IV were 12 (23.1%), 9 (17.3%), 19 (36.5%), and 12 (23.1%), respectively. The unstable healing group (types III and IV) accounted for 59.6%. Cavities were observed in 76.9% of all patients. Most of the cavities appeared as a boneless region wrapped with hardened layers. They were usually located in the anterior or middle part of the vertebral body, involving the upper endplate. In particular, some large cavities in the unstable group led to severe violation of the endplates (Figs. 4 and 5).

### Clinical manifestations

The numeric VAS score of back pain at the last follow-up did not show a significant difference between the stable and unstable groups (with an average VAS score of 0.57 points and 1.07 points, respectively), suggesting that unstable healing was painless. There was no significant difference in the ODI between patients with or without vertebral cavities (Table 2). The median ASIA impairment scale score at the final follow-up was similar between the two groups. Radiological parameters of the injured vertebra, including the Cobb angle and the average height, were not different between the two groups (data not shown). Our results suggest that most cases of unstable healing were asymptomatic.

**Table 2:** Clinical outcome measurements

	Stable Group			Unstable Group			***P***-value*
	Type I	Type II	Total	Type III	Type IV	Total	
**VAS**	0.42 ± 0.79	0.78 ± 1.20	0.57 ± 0.95	1.32 ± 1.97	0.67 ± 1.23	1.07 ± 1.7	0.247
**NDI**	2.42 ± 3.52	1.22 ± 1.92	1.91 ± 2.88	2.95 ± 4.13	2.42 ± 2.61	2.74 ± 3.52	0.382
**ASIA impairment scale**							
**B**	0	0	0	0	0	0	0.101
**C**	0	0	0	1	1	2	
**D**	0	0	0	1	3	4	
**E**	12	9	21	17	8	25	

*A *P*-value < 0.05 is considered statistically significant between the Intact Vertebra group and the Vertebral Cavity group

### Risk factors for unstable healing

To investigate the risk factors for unstable healing, demographic data between the stable group and the unstable group were compared (Fig. 1). We found that the proportion of males in the stable group (47.6%) was significantly lower than that in the unstable group (83.9%, *P* < 0.05). There were more patients with a history of smoking in the unstable group (*P* < 0.05). The LSC score and all LSC subscores were significantly greater in the unstable group (*P* < 0.001). The Spearman correlation showed that sex (*r* = 0.4), AOSpine classification (*r* = 0.5), overall LSC score (*r* = 0.5), LSC comminution score (*r* = 0.6), and LSC fragmental apposition score (*r* = 0.5) were significantly correlated with the occurrence of unstable healing (all *P* < 0.01). Further logistic regression analysis showed that only the preoperative LSC comminution score predicted the occurrence of unstable healing (*P* = 0.001, *OR* = 8.4, 95% CI = 2.4–29.7) after adjusting for sex and age. These results indicate that every point increase in the preoperative LSC comminution score was linked to an average 8-fold increase in the risk of unstable healing.

## Discussion

Instrumentation removal after vertebral healing is considered beneficial in cases of posterior short-segment fixation without fusion [9, 10]. However, its indication is unclear because the vertebra may recollapse after implant removal. In this study, we developed an HPC to estimate the stability of the healed vertebra, which is based on the size and location of cavities within the healed vertebra. Previous biomechanical studies of cadavers and finite element models have revealed that boneless defects larger than approximately 1/3 of the vertebra may significantly affect the stability of the vertebra [16, 28]. Although Costa’s finite element models were designed to simulate lytic metastatic lesions, the vertebral bodies were modeled as homogeneous and isotropic materials, while the simulated lytic lesions were modeled as spherical holes [16]. These models did not embrace the osteolytic effects that are particularly notable in metastatic diseases; thus, their conclusions can be applied directly to patients with nonmetastatic diseases.

Accordingly, we considered HPC type I and type II, in which the vertebra has no cavity or has a cavity that is smaller than 1/3 of the vertebral body volume, to indicate stable healing. Vertebrae showing these types of healing tend to have sound structural properties and are unlikely to collapse again in the future. Hence, we suggest that instruments can be removed in cases of HPC type I and type II healing.

In cases of type III healing, the cavity is larger than 1/3 of the vertebral body volume but violates no more than one endplate. In this situation, the vertebra is considered unstable, conforming to the biomechanical models. Surgeons should use their discretion and not remove the implants hastily. The large cavity may keep healing and turn into a small cavity due to the dynamic nature of the healing process. We suggest that implants in cases of type III healing should not be removed until type II healing is achieved.

The location of the lytic lesion is another factor that affects the stability of the vertebra [16]. Transcortical lesions in particular cause a significant decrease in the strength of the vertebra [29]. Correspondingly, a vertebra with a large cavity that violates both endplates or the lateral cortical shell is considered significantly unstable. We classify this healing type as HPC type IV, and we do not recommend implant removal in this situation.

This classification is simple and easy to use in clinical practice. It has excellent intraobserver and interobserver agreement, according to our results. With this classification, approximately half of the healing vertebrae in our cohort were considered unstable. This condition is easy to ignore due to the asymptomatic character of unstable healing. Doctors should keep this in mind and perform CT or MR scans before considering implant removal.

Several preoperative parameters, including the LSC and its subscores, were associated with unstable healing in our cohort. Nevertheless, only the LSC comminution score was a significant predictor according to our adjusted logistic regression model. The LSC is a three-item scale originally described in 1994 by McCormack et al. to predict the failure of short-segment fixation for traumatic thoracolumbar burst fractures [22]. At that time, they found that patients with LSC scores > 7 points were more likely to experience fixation failure. As spinal stabilization systems improved, modern posterior short-segment fixation was developed in combination with the use of intermediate screws, which was proven to have a lower implant failure rate [5, 20, 21]. Researchers further discovered that reduction can be satisfactorily achieved and maintained using this technique in patients with LSC score > 7 points [4, 30]. Thereafter, the clinical importance of the LSC became controversial. Here, we show that the preoperative LSC comminution score can predict the healing pattern outcome of the vertebra. The LSC comminution score reflects the severity of the comminution of the body on reconstructed sagittal CT images [22]. Our results suggest that the size of the cavity is related to the severity of the comminution or involvement of the vertebra. Nonetheless, its predictive ability needs further validation before recommendation for clinical use.

Surprisingly, the LSC apposition score did not predict the occurrence of unstable healing with statistical significance. One possible explanation is that the procedure of reduction during surgery may reduce the displacement of the fracture by stretching the ligament. The kyphotic deformity item of the LSC did not correlate with the occurrence of unstable healing, suggesting that the degree of correction did not affect the vertebral healing process. These results may provide a better understanding of the healing process of vertebrae after burst fracture.

Several limitations of this study need to be mentioned. The cut-off value of this classification was selected based on the results of in vitro biomechanical models. The predictive efficacy of the HPC needs to be validated over the long term in patients after implant removal. The mixture of patients with AOSpine type A3 and A4 fractures and the inconsistency of the use of mono−/poly-axial screws in the nonfractured vertebrae may have caused bias in analyzing the healing pattern distribution. The small sample size of our cohort also hindered the discovery of predictive factors for unstable healing. Furthermore, cavities originating from Schmorl’s nodes present before injury or posttraumatic intraosseous disc herniation may be confused with those originating from incomplete healing. Even so, the HPC is applicable to cavities with different traumatic pathogeneses, as they exert similar effects on the structural properties.

## Conclusion

We developed an HPC to assess the stability of healed vertebrae after implant removal. The classification was developed based on the size and location of vertebral cavities. We suggest that implants can be removed in cases of type I and II vertebral healing, while caution should be taken and instruments should not be removed in cases of type III/IV vertebral healing due to instability. Assessing the LSC comminution score preoperatively may help to predict unstable healing after surgery. Future studies on the recollapse rate for different HPC types after implant removal may further confirm the validity of this classification.

## Data Availability

The datasets used and analyzed during the current study are available from the corresponding author on reasonable request.
